# Impact of Stiffness of Quadriceps on the Pedaling Rate of Maximal Cycling

**DOI:** 10.3390/life14080956

**Published:** 2024-07-30

**Authors:** Loi Ieong, Xindi Ni, Huisong Xie, Ye Liu

**Affiliations:** 1 School of Sport Science, Beijing Sport University, Beijing 100084, China; loi_326@hotmail.com (L.I.);; 2 School of Track and Field, Beijing Sport University, Beijing 100084, China

**Keywords:** muscle stiffness, pedaling rate, rate of force development, cycling, power output

## Abstract

Propulsive power is one of the factors that determine the performance of sprint cycling. Pedaling rate is related to power output, and stiffness is associated with improving performance in athletic tasks. Purpose: to investigate the relationship between musculoarticular stiffness and pedaling rate. Methods: twenty-two healthy, untrained male volunteers (19 ± 2 years, 175 ± 6 cm, 74 ± 16 kg) were divided into two groups after their musculoarticular (MA) stiffness was tested, and these groups were the stiffness group (SG) and compliant group (CG). A 6-s maximal cycling test was conducted in four cycling modes, which were levels 5 and 10 air-resistance, and levels 3 and 7 magnetic-resistance. Peak and average cadence, peak power output (PO_peak_), crank force (CF_peak_), peak rate of crank force development (RCFD), and the angle of peak crank force were collected. The significance of differences between the two groups for these variables was assessed using an independent samples *t*-test. Pearson product–moment correlations were calculated to analyze the relationship between MA stiffness and each performance variable. Results: the SG had significantly higher peak cadence and average cadence at level 3 magnetic-resistance, peak crank force, and peak power output at level 10 air-resistance, peak rate of crank force development at levels 5 air-resistance, 10 air-resistance, and 3 magnetic-resistance (*p* < 0.05). MA stiffness was significantly correlated with average cadence at levels 5 and 10 air-resistance, peak crank force in all 4 modes, and RCFD and peak power output at level 10 air-resistance. There were no significant relationships between MA stiffness and the angle of peak crank force in each cycling mode. Conclusion: results indicate that participants with relatively higher MA stiffness seemed to have a higher pedaling rate during a 6-s sprint cycling in these conditions. They also performed a superior crank force and rate of crank force development, producing greater power output when sprint cycling. Optimizing cycling resistance or gear ratio to enhance both RCFD and musculotendinous stiffness may be crucial for improving sprint cycling performance.

## 1. Introduction

Maximal cycling power output is a significant determinant of propulsive power in sprint cycling, which is influenced by factors such as pedaling rate, crank torque, muscle size, muscle fiber-type distribution, cycling position, and fatigue [1]. The performance of sprint cycling is governed by the interplay between propulsive power and resistance forces. The pedaling rate is a dominant influencing factor in performance, which is determined by muscle shortening velocity and excitation–relaxation kinetics [1,2,3].

The relationship between muscle shortening velocity and force production, first described by Hill, demonstrates an inverse correlation. As shortening velocity increases, force production decreases, resulting in a quadratic power–velocity relationship. During maximal sprint cycling, power output initially increases with increased pedaling rate, reaching a maximum, and then it decreases with further increases in the pedaling rate [2,4]. The pedaling rate determines the time available for muscle excitation–relaxation cycles. In maximal sprint cycling, cyclists can perform pedaling cadences of up to 155 rpm [5], where the extension–flexion phases occur within 194 to 423 milliseconds [6]. However, previous research has reported that the time to peak tension for the quadriceps femoris [7] and triceps surae [8] ranges from 121 to 400 milliseconds, with half relaxation taking up to 76 milliseconds. Martin [6] indicated that this time might not be sufficient to achieve maximal force production, and the muscle force depression during the cycle would be more significant if the muscle did not reach peak tension.

Cycling efficiency seems to be related to pedaling rate. Lucia [9] found that a relatively higher preferred cadence resulted in better efficiency in professional cyclists, whereas recreational cyclists might tend to prefer lower cadences [10]. Considering discrepancies in preferred cadence, Takaishi [11] found that the cycling skills of professional cyclists might facilitate better utilization of knee flexors at higher cadences, contributing to decreased peak pedal force and lower muscle activity for the knee extensors. Additionally, higher pedaling rates may influence fiber-type recruitment patterns, favoring type I fibers and minimizing the involvement of type II fibers [12]. In summary, the optimal pedal speed is determined by muscle shortening velocity, and may exhibit different preferred pedaling rates under the same conditions. Cycling skills might alter pedal force and muscle activity levels during cycling, leading to peak power output occurring at different pedaling rates.

Recent evidence suggests that muscle stiffness is related to sports performance, especially in the utilization of the stretch-shortening cycle (SSC). Stiffness is a term used to describe the force required to achieve a certain deformation of a structure [13]. Musculoarticular (MA) stiffness comprehensively considers the stiffness of the muscle–tendon unit, surrounding articular surfaces, ligaments, and skin [14]. MA stiffness was assessed using a free oscillation technique, which has been described as a valid and reliable method for quantifying stiffness [15,16,17]. It has been reported that stiffness is related to the efficiency of storage and a release of elastic energy during the stretch-shortening cycle movements [18,19]. Increased stiffness is associated with improved performance in athletic tasks such as jumping, hopping, sprinting, and throwing. It could facilitate improved high ground-reaction forces on impact, increased ground-contact frequency, and shorter ground-contact times [19]. Previous studies have found that relatively stiffer cyclists exhibit higher crank force and rate of crank torque development (RCTD) during 6-s sprint cycling [20]. The rate of force development has recently become a popular indicator of explosive strength in athletes, which is the increase in force or torque per unit time during an explosive muscle contraction. Thus, higher stiffness might potentially enable a higher pedal frequency through improving the efficient use of elastic muscle energy.

The present study aims to investigate the relationship between musculoarticular stiffness and pedaling rate. It has previously been observed that muscle stiffness can be improved through proper resistance training [21,22,23,24]. Therefore, our findings should provide important insights into sprint cycling performance and training, which will be of interest to cyclists and coaches.

## 2. Materials and Methods

### 2.1. Subjects

Twenty-two healthy, untrained male volunteers with no prior specialized sports training background (age: 19 ± 2 years, height: 175 ± 6 cm, weight: 74 ± 16 kg) were recruited. All subjects reported engaging in physical activities 1–3 times per week. Inclusion criteria required that participants had not suffered any injury for at least 6 months prior to data collection. While participants were familiar with cycling, they were not competitive cyclists or engaged in regular cycling training. Exclusion criteria included any history of chronic diseases, current use of medications that could affect physical performance, and engagement in professional activities with a significant physical component. Participants were instructed to refrain from intense exercise for three days before the experiment. Each participant provided written informed consent, and ethical approval was obtained from Beijing Sport University.

### 2.2. Experimental Protocol

The testing involved two sessions: a 6-s sprint cycling exercise and a musculoarticular (MA) stiffness test. During the 6-s sprint cycling exercise, participants performed on a Wattbike ergometer, and the following parameters were assessed: peak cadence, average cadence, peak power output, peak crank force, and the angle at which peak crank force occurred in four cycling modes. In the MA stiffness test session, participants’ preferred legs were assessed for maximal isometric torque and MA stiffness of the quadriceps.

### 2.3. Warm-Up

Participants were instructed to perform 10 repetitions of knee-extension exercises at each of the 5 kg, 10 kg, and 15 kg loads before the maximal isometric torque and musculoarticular (MA) stiffness tests. For the 6-s sprint-cycling exercise, participants completed a comprehensive warm-up protocol. The warm-up began with a 6-min cycling session, consisting of 3 min at level 3 air-resistance followed by 3 min at level 2 magnetic-resistance. After the initial cycling, participants performed a series of dynamic stretching exercises targeting the major muscle groups involved in cycling. Following the dynamic stretches, participants executed a submaximal 6-s sprint to familiarize themselves with the test conditions and to further prepare their bodies for the maximal effort required in the formal test.

### 2.4. Cycling Performance

A 6-s cycling performance test was conducted on a Wattbike ergometer (Wattbike, Atom: Wattbike Trainer, Firmware: Model B Monitor, England). Saddle height and handlebar position were customized for each participant based on their individual anthropometric measurements to ensure a safe, comfortable, and standardized cycling position. The saddle height was set so that when the participant stood next to the bicycle, the top of the saddle was parallel to their anterior superior iliac spine (ASIS). The handlebar position was adjusted such that when the participant was sitting on the bicycle in a riding posture, a vertical line from their elbow would extend to their knee joint. The vertical distance between the handlebar and the saddle was maintained at no more than 10 cm. Participants used the same crank length. In the current experiment, the use of this same crank length on the Wattbike ergometer allowed cycling velocity to be reflected by pedaling cadence, with higher cadence indicating higher velocity.

Participants started in a static position, with the seat in the cycling position and the preferred leg at a 90-degree downstroke. Upon a start signal, participants were instructed to ride as hard as possible for 6 s while receiving strong verbal encouragement. Four cycling modes were tested: levels 5 and 10 air-resistance (level 5: pedaling rate 130 RPM = 400 W, level 10: pedaling rate 130 RPM = 595 W), and levels 3 and 7 magnetic-resistance (level 3: pedaling rate 130 RPM = 225 W, level 7: pedaling rate 130 RPM = 810 W). The air-resistance adjustment lever regulated the airflow into the flywheel, with more airflow resulting in faster pedaling and higher resistance. The magnetic-resistance adjustment lever simulated the force of gravity for a “climbing feel”, applying a fixed resistance based on the airflow into the flywheel.

Each participant completed eight maximal trials (two trials for each mode), with at least 3 min of rest between trials and 5 min between modes to avoid fatigue. The collected data included peak cadence, average cadence, peak power output (POpeak), and peak crank force (CFpeak), which were utilized for analysis. Additionally, the peak rate of crank force development (RCFD) was calculated as a relevant index to performance for analysis. RCFD was computed as the rate of change in the crank force (CF) values (from minimum to maximum, RFD = ΔF/ΔT) for the second pedal-revolution. The experiment assessed CFpeak and RCFD on the participants’ preferred leg. The angle at which peak crank force occurred was also collected.

### 2.5. Isometric Contraction Strength

Knee extensor-strength of the participant’s preferred leg was assessed on a leg-extension dynamometer (DAVID F200, Helsinki, Finland). The participant sat in the seat with the seatback and ankle cushion adjusted for maximum comfort when exerting maximum force on the device. After familiarizing themselves with the device, participants were instructed to produce maximum isometric force with the quadriceps at a knee angle of 100 degrees as quickly as possible for approximately 5 s. Participants fastened the seat belt and held onto the handles to prevent hip extension. During the testing process, strong verbal encouragement was provided, and participants were instructed to keep their buttocks in contact with the seat and their back against the seatback. Each participant completed at least three maximal trials, with the best maximal isometric torque (MIT) used for analysis.

### 2.6. MA Stiffness

A free oscillation technique [16,25,26,27] was used to assess the unilateral musculoarticular (MA) stiffness of the quadriceps. The free oscillation technique models the human body as a damped, single-degree-of-freedom “spring-mass” system. It considers the viscoelastic properties of muscle–tendon components while eliminating the influence of unrelated tissues. The technique is based on the following second-order differential equation:

(1) $$m\frac{d^{2}x}{dt^{2}}+c\frac{dx}{dt}+kx=mg$$

where m is mass, *c* is damping coefficient, *k* is stiffness, and *g* is gravitational acceleration. This method has been widely used and validated over the past 40 years, proving to be an effective way to measure muscle–tendon unit (MTU) stiffness in vivo without requiring sophisticated equipment.

The test was also performed on the leg-extension dynamometer, with the same position as the isometric test (i.e., 100 degrees at the knee angle). The dynamometer was elevated to satisfy the experimental requirements. Participants wore a customized weight-bearing boot on the distal portion of the lower leg, and carried a load approximately 50% of their maximum isometric force. A uniaxial accelerometer (YIYANG, YSV2303S, Beijing, China) recorded the oscillations, and it was attached to the distal end of the weight-bearing boot (heel of the boot). Data were sampled at 5000 Hz and recorded on a computer using data-acquisition software (YIYANG, YSV 8004 24-bits, Beijing, China). Each participant completed three trials, which were averaged for analysis. One minute of rest was provided between each trial. Data were processed using YIYANG analysis software (HDSample v7.0, Beijing, China), utilizing a band-pass filter (0–6 Hz).

### 2.7. Statistical Analysis

Participants were divided into two groups, the stiffness group (SG) and the compliant group (CG), according to their quadriceps’ stiffness ranking. The median value of the 22 subjects’ stiffness results was calculated, and the participants were divided into groups based on this value. The highest 11 stiffness values were assigned to the SG, and the lowest 11 stiffness values were assigned to the CG. An independent-samples *t*-test was conducted to compare the two groups and ensure that they had different stiffness characteristics.

After establishing the group division, the differences in all cycling performance variables and isometric contraction variables between the two groups were assessed using independent-samples *t*-tests. The effect size (ES) was calculated to quantify the magnitude of differences between the groups, using Cohen’s d.

Pearson product–moment correlation was used to analyze the relationship between musculoarticular (MA) stiffness and each performance value. Furthermore, the correlation between the rate of crank force development (RCFD) and maximal power output for each cycling level was also examined.

For all statistical analyses, the alpha level was set at *p* < 0.05. Effect sizes were interpreted as very small (Cohen’s d < 0.2), small (0.2 ≤ Cohen’s d < 0.5), medium (0.5 < Cohen’s d < 0.8), or large (Cohen’s d ≥ 0.8).

## 3. Results

There was a significant difference in MA stiffness between the SG and the CG, with that of the SG being 36% higher than that of the CG (*p* < 0.01). In addition, there were no differences in age, height, or weight between the two groups (Table 1). Furthermore, the MIT of the SG was, significantly, 30.4% higher than that of the CG.

**Figure 1:**
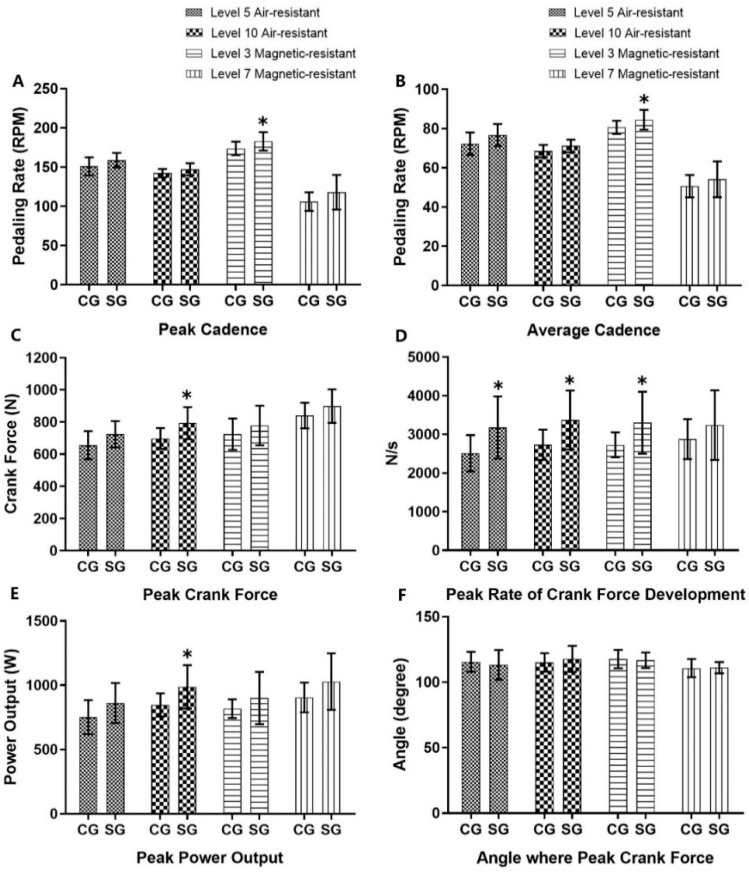
Comparison of cycling performance parameters between the compliant group (CG) and stiffness group (SG). Statistical analysis was performed using independent samples *t*-tests. (**A**) Peak cadence: SG 5.25% higher than the CG at level 3 magnetic-resistance (*p* < 0.05). (**B**) Average cadence: SG 4.73% higher than the CG at level 3 magnetic-resistance (*p* < 0.05). (**C**) Peak crank force: SG 13.7% higher than the CG at level 10 air-resistance (*p* < 0.05). (**D**) Rate of crank force development (RCFD): SG 21%, 19%, and 17.5% higher than the CG at level 5 air-resistance, level 10 air-resistance, and level 3 magnetic-resistance, respectively (all *p* < 0.05). (**E**) Peak power output: SG 14.42% higher than CG at level 10 air-resistance (*p* < 0.05). (**F**) Angle at peak crank force. * Indicates significant difference between SG and CG.

The SG reported that peak cadence, average cadence, peak crank force (CFpeak), RCFD, and peak power output (POpeak) were higher than their CG equivalents. The SG had significantly higher peak cadence (5.25%) (*p* < 0.05, Cohen d > 0.8) and average cadence (4.73%) (*p* < 0.05, Cohen d >0.8) at level 3 magnetic-resistance, peak crank force (13.7%) (*p* < 0.05, Cohen d > 0.8), and peak power output (14.42%) (*p* < 0.05, Cohen d > 0.8) at level 10 air-resistance, peak rate of crank force development (21%, 19%, 17.5%) at level 5 air-resistance (*p* < 0.05, Cohen d > 0.8), 10 air-resistance (*p* < 0.05, Cohen d > 0.8), and level 3 magnetic-resistance (*p* < 0.05, Cohen d > 0.8) (Figure 1).

**Figure 2:**
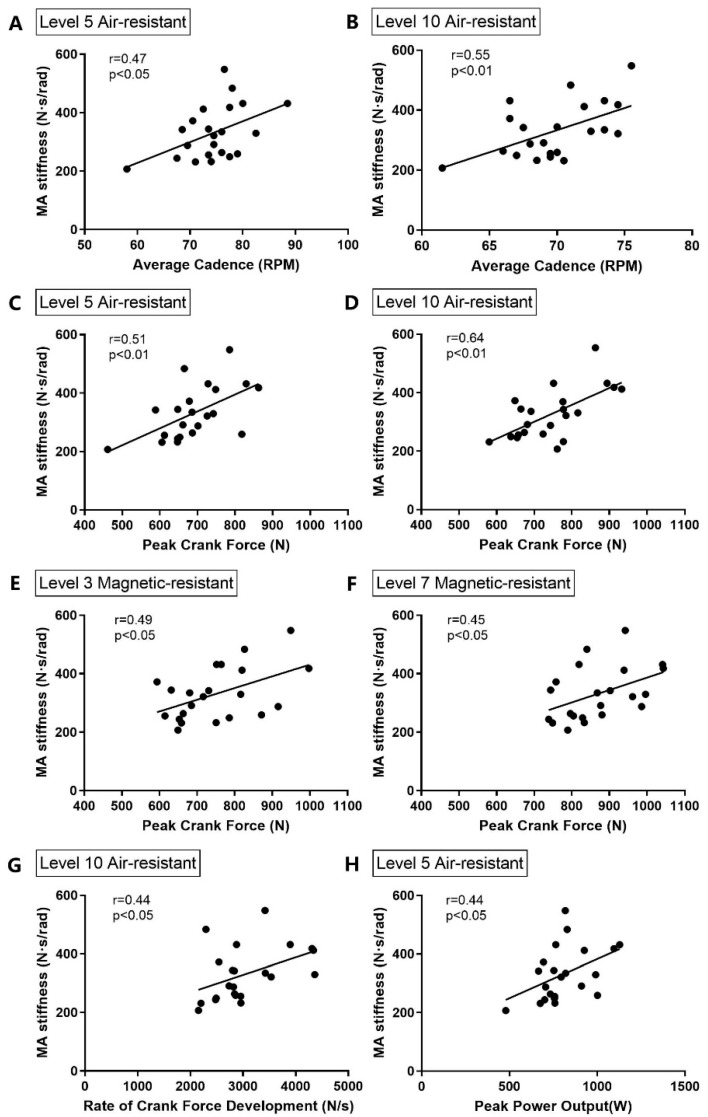
Correlations between MA stiffness and cycling performance parameters. Statistical analysis was performed using Pearson’s correlation coefficient. (**A**,**B**) Average cadence at levels 5 and 10 air-resistance. (**C**–**F**) Peak crank force at four cycling levels. (**G**) Rate of crank force development (RCFD) at level 10 air-resistance. (**H**) peak power output at level 5 air-resistance. Significant positive correlations were observed for all displayed relationships (*p* < 0.05).

MA stiffness was a significant and largely positive relationship with MIT (r = 0.88, *p* < 0.01) in isometric contraction-strength. The significant relationship between MA stiffness and average cadence during cycling at levels 5 and 10 air-resistance is displayed in Figure 2A,B. MA stiffness was significantly correlated with peak crank force during cycling at 4 cycling levels (Figure 2C–F). In addition, MA stiffness was also significantly correlated with RCFD and peak power output during cycling at level 10 air-resistance (Figure 2G). Furthermore, there were no significant relationships between MA stiffness and the angle of peak crank force during cycling. The relationship between RCFD and peak power output during cycling was found to be a largely positive, significant relationship at each cycling level, namely, r = 0.85 at level 5 air-resistance, r = 0.81 at level 10 air-resistance, r = 0.81 at level 3 magnetic-resistance, and r = 0.83 at level 7 magnetic-resistance.

## 4. Discussion

Cycling is a unique racing event that requires cyclists to exert force on the cranks of their bicycles. The performance of sprint cycling is influenced by factors such as power output, air-resistance, rolling-resistance, and bearing drag. Power output (P) is the amount of work (W) transferred per unit of time (t), represented by the equation P = W/t = F·v, where F is force and v is velocity.

Participants with relatively higher musculoarticular (MA) stiffness exhibited significantly higher peak and average cadence under the level 3 magnetic-resistance condition during the 6-s cycling sprint (Figure 1), and a trend of increased cadence was observed in the other three resistance conditions. Furthermore, there were significant, medium, positive correlations between MA stiffness and average cadence in the levels 5 (r = 0.47) and 10 (r = 0.55) air-resistance conditions. This evidence suggests that relatively stiffer musculotendinous units may be associated with higher pedaling cadence, with significant differences observed under certain conditions. This study is the first to reveal the relationship between MA stiffness and pedaling cadence.

Muscular stiffness enhances the transmission of elastic energy from tendons during muscle contraction [28]. Our study supports this view. Participants in the stiffness group (SG) exhibited higher rates of crank-force development (RCFD) during the 6-s sprint cycling, with significant differences observed in the levels 5 and 10 air-resistance, and level 3 magnetic-resistance, conditions (Figure 1). The rate of force development (RFD) is an important index characterizing explosive strength, which is the capacity to produce maximal voluntary activation in the early phase of explosive contractions [22]. RCFD is a specific application in cycling, computed as the rate of change in crank force (CF) values. An efficient pedaling stroke depends on the stretch-shortening cycle (SSC). During the upstroke, the quadriceps contracts eccentrically for knee flexion, followed by a concentric contraction for knee extension during the downstroke. Cyclists with relatively higher musculoarticular (MA) stiffness may enhance concentric performance by better utilizing stored elastic energy from the eccentric phase [18,19,20] during the SSC movement, thereby increasing muscle contraction velocity [29]. Furthermore, it has been reported that the series elastic element could increase the maximum fiber shortening velocity and reduce tendon slack length [29]. Previous studies have noted a correlation between tendon slack length and electromechanical delay (EMD) [30], indicating that a lower EMD in higher MA stiffness could reduce the time required to stretch the series elastic component, improving force transmission from the contractile element to the bone, and thus increasing RFD [31]. Consequently, higher MA stiffness may contribute to superior RCFD performance, enhancing pedaling rate during 6-s sprint cycling.

The results of this study also revealed a significant moderate correlation between RCFD and MA stiffness (r = 0.44, *p* < 0.05). These findings support the review by Maffiuletti [22], which suggested that an increase in RFD with training-induced adaptations may be related to changes in musculotendinous stiffness, likely affecting the capacity for rapid force increases at the onset of contraction [31]. The initial acceleration response is critical for sprint cyclists, and it may be improved by enhancing RCFD. Accordingly, selecting an appropriate cycling resistance or gear ratio to improve both RCFD and musculotendinous stiffness might be crucial. Future research is needed to determine whether increasing RCFD influences MA stiffness, and whether different loads used to achieve higher speed-training influences MA stiffness.

Furthermore, there was no difference in the angle at which peak crank force occurred between the compliant group (CG) and the stiffness group (SG), and no correlation between the angle at which peak crank force occurred and MA stiffness. This result is inconsistent with Watsford [20], who observed a negative correlation between MA stiffness and the angle at which peak crank force occurred, with a lower angle of peak crank force occurring in higher MA stiffness. The possible reasons are as follows. Firstly, there are differences in the experimental design. In this experiment, the pedaling cadence during cycling was not fixed, while Watsford adopted a fixed frequency of 80 rpm. Whether the pedaling cadence is fixed or not may affect the force-generation pattern and energy output of the muscles, and subsequently may influence the angle at which the peak crank force occurs. Secondly, the selection of participants varies. The experiment selected trained male cyclists, while this experiment chose male college students without systematic training experience. These untrained male college students might have had difficulty maintaining a stable force application and rhythm during cycling, which could have made the angle at which the peak crank force occurs more random, thereby resulting in a difference from Watsford’s experimental results.

Relatively stiffer musculotendinous units have a higher capacity for force or torque production [32,33,34]. In accordance with previous studies [16,17,20], our results showed that the SG exhibited significantly higher maximal isometric torque (MIT), and MIT was significantly, largely correlated with MA stiffness. Wilson [35] assumed that higher MA stiffness displays a better optimal resting length of the muscle. The popular Hill three-component model [36] depicts muscle as consisting of a contractile component (CC), serial elastic components (SEC), and parallel elastic components (PEC). When the CC is inactive or has low activity, the tension of the musculotendinous unit is borne by the PEC, consisting of muscle fasciae, sarcolemma, and interactions between filaments and residual cross-bridge attachments [37]. Based on the sarcomere length–tension relation of muscle, maximal contraction strength is achieved at the optimal resting length. A greater optimal resting length may promote a relatively higher force output.

Musculoarticular (MA) stiffness also appears to be related to power output during 6-s sprint cycling. The results of this study show that the stiffness group (SG) exhibited higher peak power output (POpeak) than the compliant group (CG), with a significantly larger difference observed in the level 10 air-resistance condition. Cadence and power output represent riding velocity, and the combination of forces and velocity during the 6-s sprint cycling, respectively. Cycling performance is determined by the sources of resistance and the rider’s power output, which includes factors such as riding position, body mass, rolling resistance, air-resistance, and the individual’s aerobic and anaerobic power and capacity for skeletal muscle contraction. Our research suggests a relative relationship between MA stiffness and cadence, and power output in sprint cycling. The cycling cadence and power output represent the overall performance, incorporating physical, physiological, and cycling technique elements of sprint cyclists. Sprint cyclists with higher MA stiffness might have a greater optimal resting length and a shorter electromechanical delay (EMD) to produce higher force output and rate of crank force development (RCFD), allowing them to store more energy for pedal strokes with higher peak crank force (CFpeak), resulting in higher cadence and power output. Our findings also revealed that RCFD was significantly, largely positively, correlated with POpeak in each cycling mode, and the average cadence and POpeak were significantly, moderately positively, correlated with MA stiffness at levels 5 and 10 air-resistance, and level 5 air-resistance, respectively. This suggests that modifying musculotendinous stiffness may be considered to improve sprint cycling performance. Foure [38] and Kubo [39,40] indicated that the stiffness of the muscle–tendon complex increased after long-term plyometric, isometric, and weight training. However, high levels of stiffness may be associated with increased peak forces, impact forces, and reduced joint motion, increasing the risk of bony injuries [19,41].

The resistant mode was considered in the selection of cycling resistance for our experiment. In the level 3 magnetic-resistance condition, both the SG and CG exhibited the highest peak and average pedaling cadences, and significant differences were reported between the two groups. The difference between the two resistance modes is that the cycling resistance varies with cycling frequency for air-resistance but not for magnetic-resistance. The change in magnetic-resistance is smaller than that in air-resistance. This might better reflect the pedaling cadence performance of sprint cyclists under conditions of lower initial resistance and smaller frequency-dependent changes. However, when cycling at the highest resistance (level 7 magnetic-resistance), there were no significant differences in any parameter between the two groups. This lack of difference at high resistance may be attributed to the substantial resistance requiring subjects to recruit more muscle fibers to generate sufficient force to drive the crank. Consequently, the explosive power advantage of the high-stiffness group may have been diminished. Furthermore, significant differences between the two stiffness groups were observed in maximum peak force and maximum power at air-resistance level 10. The difference in maximum force-loading rate was also more pronounced at air-resistance level 10, compared to air-resistance level 5 and magnetic-resistance level 3. These findings may be explained by the variable nature of air-resistance, where pedaling force and power change with pedaling speed. In contrast, magnetic-resistance provides a fixed resistance, requiring subjects to exert a consistently high force to drive the crank. Therefore, the air-resistance mode may better reflect the explosive power capacity of cyclists. Our results suggest that the air-resistance mode may be more suitable for developing cyclists’ explosive power ability, while the magnetic-resistance mode may be more appropriate for improving speed endurance capabilities. Researchers should consider these findings when selecting the appropriate resistance mode for their experimental needs.

Several limitations of this study should be acknowledged. Firstly, from a physiological perspective, individuals with higher body weight typically have greater muscle mass, which may influence joint stiffness. Future research could increase sample size and employ statistical methods to mitigate the impact of body weight on results, thereby more accurately assessing the relationships between target variables. Secondly, our study primarily focused on the ‘anatomical’ or inherent stiffness of the muscle–tendon unit, without directly measuring or controlling for the effects of voluntary modulation of joint stiffness through neural control and muscle co-activation. Recent literature [42,43] has indicated that joint stiffness can be voluntarily modulated through the co-activation of antagonist muscles. This neural control aspect could potentially impact our results, as athletes might compensate for lower inherent stiffness through more efficient neural control and muscle co-ordination.

## 5. Conclusions

This study suggests that participants with relatively higher musculoarticular (MA) stiffness demonstrated higher pedaling rates during 6-s sprint cycling conditions. These participants also exhibited superior crank force and rate of crank force development, resulting in greater power output during sprint cycling. However, there was no correlation observed between the angle at which peak crank force occurred and MA stiffness. These findings suggest that selecting an appropriate cycling resistance or gear ratio to improve both rate of crank force development (RCFD) and musculotendinous stiffness might be crucial for enhancing sprint cycling performance.

## Figures and Tables

**Table 1:** Summary of the parameters (±SD) for anthropometric stiffness and isometric torque.

Basic Variables	All Subjects (*n* = 22)	CG (*n* = 11)	SG (*n* = 11)	*p*	ES (Cohen d)
Age (year)	19 ± 2	20 ± 3	19 ± 1	0.44	0.34
Height (cm)	175 ± 6	175 ± 7	175 ± 4	0.97	0.02
Weight (kg)	74 ± 16	68 ± 12	79 ± 17	0.09	0.75
MA stiffness	331.52 ± 91.24	258.60 ± 32.05	404.43 ± 68.98 *	<0.01	2.7
MIT	212.82 ± 53.88	174.64 ± 27.18	251 ± 46.36 *	<0.01	2.01

* Significant difference from the CG.

## Data Availability

The data presented in this study are available on request from the corresponding author. The data are not publicly available due to privacy and ethical restrictions, as they contain information that could compromise the privacy of research participants.
